# Comparisons of Differentiation Potential in Human Mesenchymal Stem Cells from Wharton's Jelly, Bone Marrow, and Pancreatic Tissues

**DOI:** 10.1155/2015/306158

**Published:** 2015-07-29

**Authors:** Shih-Yi Kao, Jia-Fwu Shyu, Hwai-Shi Wang, Chi-Hung Lin, Cheng-Hsi Su, Tien-Hua Chen, Zen-Chung Weng, Pei-Jiun Tsai

**Affiliations:** ^1^Ten-Chan General Hospital Zhongli, Taoyuan City 112, Taiwan; ^2^Department of Biology and Anatomy, National Defense Medical Center, Taipei 112, Taiwan; ^3^Institute of Anatomy and Cell Biology, School of Medicine, National Yang-Ming University, Taipei 112, Taiwan; ^4^Institute of Clinical Medicine, National Yang-Ming University, Taipei 112, Taiwan; ^5^Institute of Microbiology and Immunology, National Yang-Ming University, Taipei 112, Taiwan; ^6^Department of Surgery, Cheng Hsin General Hospital, Taipei 112, Taiwan; ^7^Department of Surgery, Taipei Veterans General Hospital, Taipei 112, Taiwan; ^8^Division of Cardiovascular Surgery, Department of Surgery, Taipei Medical University Hospital, Taipei 112, Taiwan; ^9^Department of Surgery, School of Medicine, College of Medicine, Taipei Medical University, Taipei 112, Taiwan; ^10^Department of Critical Care Medicine, Taipei Veterans General Hospital, Taipei 112, Taiwan

## Abstract

*Background*. Type 1 diabetes mellitus results from autoimmune destruction of *β*-cells. Insulin-producing cells (IPCs) differentiated from mesenchymal stem cells (MSCs) in human tissues decrease blood glucose levels and improve survival in diabetic rats. We compared the differential ability and the curative effect of IPCs from three types of human tissue to determine the ideal source of cell therapy for diabetes. *Methods*. We induced MSCs from Wharton's jelly (WJ), bone marrow (BM), and surgically resected pancreatic tissue to differentiate into IPCs. The *in vitro* differential function of these IPCs was compared by insulin-to-DNA ratios and C-peptide levels after glucose challenge. *In vivo* curative effects of IPCs transplanted into diabetic rats were monitored by weekly blood glucose measurement. *Results*. WJ-MSCs showed better proliferation and differentiation potential than pancreatic MSCs and BM-MSCs. *In vivo*, WJ-IPCs significantly reduced blood glucose levels at first week after transplantation and maintained significant decrease till week 8. BM-IPCs reduced blood glucose levels at first week but gradually increased since week 3. In resected pancreas-IPCs group, blood glucose levels were significantly reduced till two weeks after transplantation and gradually increased since week 4. *Conclusion*. WJ-MSCs are the most promising stem cell source for *β*-cell regeneration in diabetes treatment.

## 1. Introduction

Diabetes mellitus (DM) is one of the leading causes of death in the world and more and more people suffer from this widespread disease [1, 2]. It has been recognized that Type 1 DM, called juvenile-onset diabetes, develops due to beta cells being attacked and destroyed by the individual's own immune system. Type 2 DM, adult-onset diabetes, is characterized by insulin resistance, resulting in the ineffectiveness of insulin [3]. Both types of DM lead to rising blood glucose levels, which are associated with many complications such as retinopathy, nephropathy, and neuropathy, among others [4]. Subcutaneous injections of insulin are commonly used to manage Type 1 DM as well as the later stage of Type 2 DM. However, the requirements of frequent insulin injections and troublesome blood glucose monitoring have been criticized, as well as the lack of cure for diabetes [5].

Transplantation of cadaveric pancreases began to flourish in 1966, allowing people with diabetes to live without insulin injections [6]. As the success rate of transplantation increased, it became widely popular. However, disadvantages were noted, including shortage of donor pancreases, a certain degree of surgical risk, and high risk of complications after patients received life-long immunosuppression. Therefore, pancreas islet transplantation emerged as another possible solution for diabetes. Currently, the most effective protocol is the Edmonton protocol, which involves a one-time injection of* in vivo* expanded islet cells from at least two donors via the portal vein. Though the procedure reduces surgical risks, its therapeutic effect can only be sustained for about 10 years with an insulin independence rate of not more than 15%. Also, the demand for donor islets still outweighs their availability. Meanwhile, long-term use of immunosuppressants is still necessary accompanied by its inevitable side effects [7].

In recent years, researchers have made great efforts to develop regeneration therapy, in which stem cells or endocrine precursor cells are stimulated to differentiate into insulin-producing cells (IPCs) for replacing destroyed *β* cells. Regeneration therapy progresses rapidly because it has potentially fewer limitations in comparison to the above two therapeutic strategies [8].

In general, the ideal tissue source for regeneration therapy for diabetes must meet certain criteria such as abundant availability, easy duplication, and equivalent function to that of the primary beta cell. Not only embryonic stem cells, but also adult stem cells, adult human pancreatic precursor cells, and extrapancreatic endocrine progenitor cells have been reported as surrogate *β*-cells in the literature [8, 9]. Although great advances have been achieved in generating *β*-cells, there is not yet any consensus on what kind of cell source best meets the requirements for treating diabetes.

Our laboratory has demonstrated that pancreatic endocrine precursor (PEP) cells can be generated from Wharton's jelly mesenchymal stem cells (WJ-MSCs) [10, 11], bone marrow mesenchymal stem cells (BM-MSCs) [12], and surgically resected adult pancreatic tissues [13]. PEP cells can be induced into IPCs and are able to reverse hyperglycemia after transplantation in STZ-induced diabetic rats. The aim of this study was to compare the* in vitro* differential ability and the* in vivo* curative effect of IPCs generated from different sources, including Wharton's jelly, BM, and pancreatic tissues, to determine the ideal source of cell therapy for treatment of diabetes.

## 2. Methods

### 2.1. Isolation and Differentiation of IPCs from Resected Human Pancreatic Tissue

Institutional Review Board approval (Taipei Veterans General Hospital) was obtained for all procedures. With the written informed consent of the parents, the healthy pancreatic parenchyma tissue was resected from the normal portion which was used for anastomosis. To prevent degradation, the fresh pancreatic tissue was initially preserved in solution D (0.137 M NaCl, 5.38 mM KCl, 0.19 mM Na_2_HPO_4_, 0.205 mM K_2_HPO_4_, 5.49 mM glucose, 0.058 M sucrose, 1% penicillin/streptomycin, and 0.12% fungizone). The tissue was then minced and digested by 2 mg/mL Type V collagenase (Sigma-Aldrich, St. Louis, MO) for 30 min at 37°C. The digested sample was washed three times with cold Dulbecco's modified Eagle medium/F12 (DMEM/F12, Invitrogen, Carlsbad, CA). After centrifugation at 1200 g for 20 minutes at 4°C in Histopaque (1.077 mg/mL) and DMEM/F12 gradients, pancreatic duct cells, islets, and endocrine precursor cells (EPCs) were isolated. The EPCs from the Histopaque/DMEM interface were aspirated and washed with DMEM/F12 and then cultured with CMRL 1066 medium (5.5 mM glucose, Invitrogen corporation) containing 10% FBS, 1% penicillin/streptomycin, 100 ng/mL nerve growth factor (R&D Systems, Minneapolis, MN), 10 mM nicotinamide (Sigma), and 25 ng/mL epidermal growth factor (EGF, Invitrogen). After 7–10 expansion days, the EPCs reached confluence. The EPCs were trypsinized with 0.05% trypsin/EDTA (Invitrogen), washed with serum-free DMEM/F12 (17.5 mmol/l glucose), and seeded into 6-well culture dishes coated with Matrigel (BD Bioscience, Bedford, MA, USA) for further culture and differentiation. The number of the EPCSs in each well was 1 × 10^6^ cells. Insulin, transferrin, sodium selenite + linoleic acid (ITS + l, Sigma), 2 g/L BSA, and 10 ng/mL basic fibroblastic growth factor (bFGF, Invitrogen) were added in the culture medium. After 5–7 days in Matrigel, the cells aggregated from monolayers to clusters and differentiated into IPCs. The gel layer was then disrupted with a cell scraper. Both the IPC clusters and the Matrigel pieces were transferred to a large volume of prewarmed medium and individual cell clusters were handpicked with a fire-polished glass pipette. The IPC clusters were then kept in suspension 5 days in serum-free DMEM/F12 supplemented with ITS + l [13].

### 2.2. Isolation and Differentiation of IPCs from BM-MSCs

All study procedures were approved by the Institutional Review Board (Taipei Veterans General Hospital). Bone marrow tissues were gathered from 20 healthy donors with their informed consent. After washing the bone marrow sample twice with phosphate buffered saline (PBS, PH = 7.2), density gradient centrifugation (NycoPrep 1.077, Axis-Shield, Oslo, Norway) was possessed and BM-MSCs were isolated. Rinse the BM-MSCs twice in low glucose DMEM (LG-DMEM, 5.5 mM glucose, Invitrogen, Carlsbad, CA) and culture them at 37°C with 5% humidified CO2 in expansion medium consisting of L-DMEM supplemented with 10% fetal bovine serum (FBS; Invitrogen) and 1% penicillin/streptomycin/Amphotericin (Biological Industries, Haifa, Israel). The culture medium was replaced every 3 days and the nonadherent cells were removed. When the adherent BM-MSCs were 90–95% confluent (10–15 days), they were subcultured by Trypsin-Versene (Invitrogen). When the third passage BM-MSC reached 80% confluence, it was provided to differentiate into IPCs by culturing in serum-free high glucose DMEM (HG-DMEM, 25 mM glucose) supplemented with 1% dimethyl sulfoxide (DMSO, Sigma, St. Louis, MO). 3 days after, the culture medium was replaced with HG-DMEM supplemented with 10% FBS for another 14 days [12].

### 2.3. Isolation and Differentiation of IPCs from WJ-MSCs

All study procedures were approved by the Institutional Review Board (Taipei Veterans General Hospital). With the written informed consent of the parents, fresh human umbilical cords were obtained after birth and stored in Hank's balanced salt solution (Biological Industries, Israel) prior to tissue processing to obtain MSCs. After removal of blood vessels, the mesenchymal tissue was scraped off the Wharton's jelly and centrifuged at 250 g for 5 min. After centrifugation, the pellets were resuspended in 15 mL of serum-free Dulbecco's modified Eagle's medium (DMEM; Gibco, Grand Island, NY) containing 0.2 g/mL of collagenase and incubated for 16 h at 37°C. Next, the cells were washed, resuspended in DMEM containing 2.5% trypsin, and incubated for 30 min at 37°C with agitation. Finally, cells were again washed and cultured in DMEM supplemented with 10% fetal bovine serum (FBS; Sigma St. Louis, MO, USA) and glucose (4.5 g/L) in 5% CO_2_ in a 37°C incubator. At the fourth to sixth passage, after reaching a confluence of 70%, the MSCs were induced to differentiate into islet-like cell aggregates with three stages. Undifferentiated MSCs were detached by HyQTase, diluted with SFM-A, and centrifuged. Cells were counted for initial seeding density and 1 × 10^6^ cells/cm^2^ were resuspended in SFM-A and seeded on ultralow attachment tissue culture plates (Corning, Fisher Scientific International, Hampton, NH, http://www.fisherscientific.com/). SFM-A contained DMEM/F12 (1 : 1) (Gibco, Grand Island, NY) with 17.5 Mm glucose, 1% BSA Cohn fraction V, fatty acid free (Sigma-Aldrich), 1% penicillin/streptomycin/amphoteric B (PSA; Biological Industries, Israel), insulin-transferrin-selenium-X (ITS-X; 5 mg/L insulin, 5 mg/L transferrin, 5 mg/L selenium), 4 nM activin A, 1 mM sodium butyrate, and 50 *μ*M 2-mercaptoethanol. The cells were cultured in this medium for 2 days. On the third day, the culture medium was changed to SFM-B, which contains DMEM/F12 (1 : 1) with 17.5 mM glucose, 1% BSA, 1% PSA, ITS-X, and 0.3 mM taurine. On the fifth day, the cell culture was replaced by SFM-C, which contained DMEM/F12 (1 : 1) with 17.5 mM glucose, 1.5% BSA, ITS-X, 1% PSA, 3 mM taurine, 100 nM glucagon-like peptide (GLP)-1 (amide fragment 7–36; Sigma Aldrich), 1 mM nicotinamide, and nonessential amino acids (NEAAs). For the next 5 days, the culture medium was exchanged with fresh SFM-C every 2 days [11].

### 2.4. Reverse Transcriptase-Polymerase Chain Reaction (RT-PCR) and Real-Time PCR Analysis to Determine Gene Expressions in Differentiated Cells

To determine whether the three kinds of cell sources had differentiated into IPCs, the expressions of genes involved with both pancreatic *β*-cell development and insulin production were examined by RT-PCR and real-time PCR. All RNA extractions from the three types of differentiated cells were performed with Trizol reagent (Invitrogen) according to the manufacturer's instructions. cDNA was prepared from 4 mg RNA using the Superscript TM III first-strand synthesis system (Invitrogen). PCR was performed with 200 ng RNA equivalents using specific primers in the presence of SYBR Green I (LightCycler TM-FastStart DNA Master SYBR Green I; Roche, Basel, Switzerland). Primers were Pdx1 forward GGAGCCGGAGGAGAACAAG, reverse CTCGGTCAAGTTCAACATGACAG; Pax4 forward GGGTCTGGTTTTCCAACAGAAG, reverse CAGCGCTGCTGGACTT; Glut2 forward GCCTAGTTATGCATGCAG, reverse GGTTTGTAACTTATGCCTAAG; insulin forward ACCAGCATCTGCTCCCTCTA, reverse GGTTCAAGGGCTTTATTCCA; GAPDH forward CACCATCTTCCAGGAGCGAG, reverse TCACGCCACAGTTTCCCGGA (Mission Biotech, Taiwan). A LightCycler 480 (Roche, Indianapolis, IN) was used for real-time PCR with the following cycling program: 50°C for 2 min, 95°C for 10 min, and 35 cycles at 95°C for 15 s and 60°C for 1 min. Melting curves were obtained at 60°C. The number of PCR cycles was titrated in order to remain in the linear range of amplification. The resultant amplification products (10 mL) were separated using 2% agarose gel electrophoresis and were visualized with ethidium bromide that validate the specificity of the real-time PCRs [11–13].

### 2.5. Measurement of Insulin-to-DNA Ratio

The three types of differentiated cells were washed twice with PBS, resuspended in 300 mL of distilled cold water, and homogenized by sonication on ice. An aliquot of the homogenates was analyzed fluorometrically for DNA content in duplicate, and another aliquot was extracted with acid ethanol overnight and measured for insulin content using an ELISA kit (Mercodia, Uppsala, Sweden).

### 2.6. Measurement of C-Peptide Level after Glucose Challenge Test

The three types of differentiated cells were incubated for 1 h in DMEM-LG (5.5 mM glucose) and the medium was collected and stored at −20°C. The cells were washed with PBS and incubated for 1 h in DMEM-HG (25 mM glucose) (Gibco, NY) and the medium was collected and stored at −20°C. The C-peptide concentration was determined by C-peptide ELISA kit (Mercodia, Uppsala, Sweden).

### 2.7. Comparison of* In Vivo* Curative Effect by Intrahepatic IPCs Injection in STZ-Induced Diabetic Rats

Hyperglycemia was induced in 24 male SD rats of closed colony (body weight 300–350 g) through intraperitoneal injection of 30 mg/kg of streptozotocin (STZ) on 3 consecutive days. Blood glucose levels were determined using Roche ACCU-CHEK glucose meter (Roche Diagnostics, Indianapolis, IN, USA.) by tapped tail-vein blood. Stable hyperglycemia (blood glucose levels ranging between 16.7 and 33.3 mmol/L) developed in 24 rats one week later. The 18 diabetic rats, 6 in each of the three study groups, were anesthetized with pentobarbital (40 mg/kg, i.p.). After midline laparotomy, the portal vein was identified and 0.5 mL heparinized saline and 5 × 10^6^ differentiated insulin-producing cells (three types of differentiated cells) suspended in 0.1 mL of normal saline were injected into the catheters of WJ-MSCs, BM-MSCs, and pancreatic MSCs study groups, followed by a volume of normal saline equivalent to the volume of the Port-A-Cath catheter (0.35 mL) to push the grafts into the portal vein. The 6 rats in the STZ group underwent the same procedure but were only injected with normal saline (STZ group). Body weight and blood sugar levels were recorded before and after cell transplantation. Blood was collected from a tail vein and blood glucose levels were measured with a blood glucose meter (Roche, Basel, Switzerland) [11–13].

### 2.8. Immunofluorescence Analysis

The rats were sacrificed 8 weeks after transplantation and perfused with 4% formaldehyde (Ferak, Berlin, Germany). The pancreatic tissues were resected and cut into 0.5–1.0 cm^3^ pieces. The samples were dehydrated and embedded in OCT (Sakura Finetek USA Inc., Torrance, CA, USA) in liquid nitrogen. The cryosections (5 *μ*m/piece) were washed twice with PBS and incubated overnight at 4°C with rabbit anti-human C-peptide antibodies (1 : 100; Santa Cruz, Santa Cruz, CA, USA). After 3 washes in PBS, slides were incubated for 1 h at room temperature with Cy3-labeled goat anti-rabbit IgG (1 : 200, Jackson ImmunoResearch, West Grove, PA, USA). Nuclei were counterstained using DAPI (1 : 5000, Molecular Probes, Inc., Eugene, OR, USA). After the sections were mounted with mounting medium (Vector Laboratories, Burlingame, CA, USA), microscopy was performed using a confocal microscope equipped with difference interference contrast light path (LSM 510, Zeiss, Göttingen, Germany).

### 2.9. Statistical Analysis

Each series of experiments was performed in triplicate. The results obtained from a typical experiment were expressed as the means ± standard deviation (SD). Statistical analysis was carried out using the SPSS 14.0 software program (Statistics Package for Social Sciences, SPSS Inc. Chicago, IL, USA). Statistical analysis used nonparametric Mann-Whitney* U* test (2 independent samples). A *P* value of less than, or equal to, 0.05 was established as statistical significance.

## 3. Results

### 3.1. Isolation, Cultivation, and Differentiation of Resected Human Pancreatic Tissue, Human BM-MSCs, and WJ-MSCs (See Figure 5)

**Figure 1:**
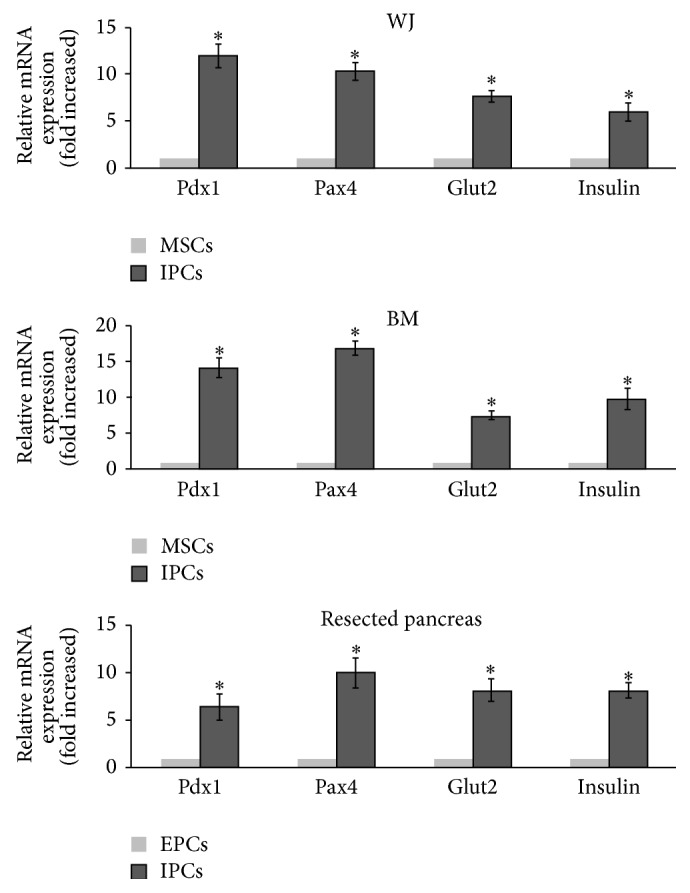
Real-time PCR analyses of three kinds of cell sources after differentiation to evaluate the expression of pancreatic *β*-cell development-related and insulin production-related genes, including Pdx1, Pax4, Glut2, and Insulin. Results are the means ± SD for 6 experiments. ^∗^:*P* < 0.05 compared to nondifferentiated cells.

To determine whether the three kinds of cell sources had differentiated into IPCs, the expression of genes involved in pancreatic *β*-cell development and insulin production was examined by reverse transcriptase-PCR and real-time PCR. As shown in Figure 1, the pancreatic *β*-cell development related genes, including Pdx1, Pax4, Glut2, and insulin, were significantly expressed in differentiated IPCs greater than in undifferentiated cells from the three kinds of cell sources. It was a guarantee that they were real IPCs after our differentiation procedures. Besides, as described in the previously published articles, surgical pancreatic MSCs stained positive for C-peptide after 23 days of culturing in differentiation medium with an overall culture success rate of 35% and differentiation potential of only one passage [13]. BM-MSCs stained positive for C-peptide at days 14 and 18 of differentiation, with greater expression on day 14 and differentiation potential of 4–6 passages [12]. WJ-MSCs stained with anti-human C-peptide antibodies showed that C-peptide was expressed at days 5 and 10 of differentiation, with greater expression on day 10 and differentiation potential of 8–10 passages [11].

### 3.2. Detection of Insulin/DNA Ratio in Differentiated Cells Derived from Resected Human Pancreatic Tissue, Human BM-MSCs, and WJ-MSCs

**Figure 2:**
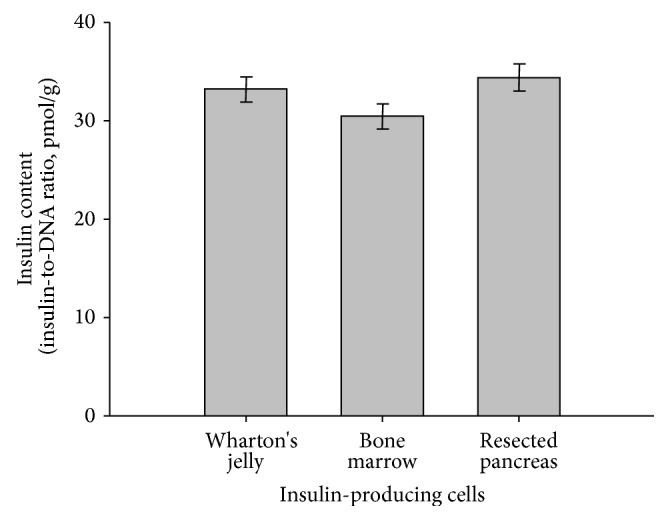
Insulin-to-DNA ratio in three different types of human tissue MSCs. Insulin-producing cells cultured in the maturation phase were stimulated as indicated and C-peptide in the medium was analyzed using ELISA. Measurements were normalized for DNA content of each sample. Three samples per condition were measured. Similar results were obtained in at least three independent experiments.

The expression of insulin/DNA ratio was greatest in human resected pancreas-IPCs compared to human WJ -IPCs and BM-IPCs. The expression of insulin/DNA ratio was greater in WJ-IPCs than in BM-IPCs. However, no significant differences were found in above comparisons (Figure 2).

### 3.3. Comparison of C-Peptide Secretion by Differentiated Cells in Response to Glucose Stimulation

**Figure 3:**
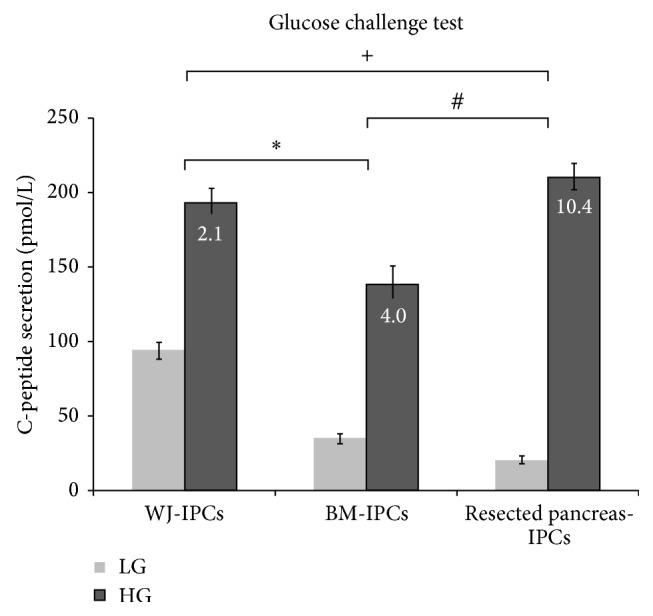
Secretion of C-peptide by cultured human IPCs in response to glucose stimulation. Glucose challenge test for C-peptide release in response to low glucose (LG: 5.5 mM) and high glucose (HG: 25 mM) concentrations of differentiated cells. (^+^: the increasing fold of resected pancreas compared to the increasing fold of Wharton's jelly, *P* < 0.05; ^#^: the increasing fold of resected pancreas compared to the increasing fold of bone marrow, *P* < 0.05; ^∗^: the increasing fold of bone marrow compared to the increasing fold of Wharton's jelly).

To test whether the three kinds of IPCs have functional characteristics of pancreatic beta cells, we determined the secretion of C-peptide by each kind of IPCs at both low glucose concentration (5.5 mM) and high glucose concentration (25 mM). At a low glucose concentration, the IPCs from WJ-MCSs showed the highest release of C-peptide compared to IPCs from BM-MCSs and resected pancreas EPCs. At a high glucose concentration, the IPCs from WJ-MCSs showed the similar release of C-peptide as IPCs from BM-MCSs, both of which were higher than IPCs from resected pancreas EPCs. On the other hand, all of the three kinds of IPCs could significantly increase the C-peptide secretion in response to higher glucose stimulation. IPCs from the resected pancreas EPCs showed at least 10-fold increases in stimulated C-peptide secretion in response to high glucose. The IPCs from BM-MSCs revealed at least 4-fold increases in stimulated C-peptide secretion in response to high glucose. The IPCs from WJ-MSCs produced approximately twice the amount of C-peptide secretion at a high glucose concentration (Figure 3).

### 3.4. Changes in Blood Glucose in Rats after Transplantation of IPCs Derived from Resected Pancreas, BM-MSCs, and WJ-MSCs

**Figure 4:**
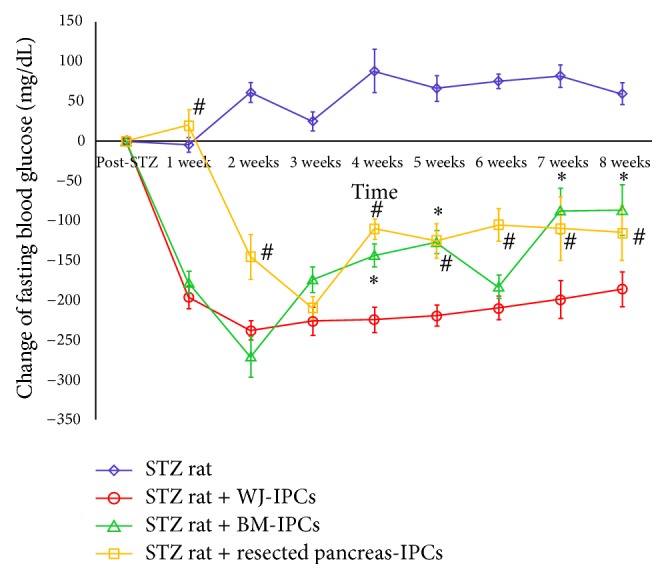
Changes in blood glucose levels in STZ-induced diabetic rats (study group: transplantation of IPCs into the portal vein via the Port-A-Cath with Wharton's jelly MSCs, BM-MSCs, and resected pancreas; STZ group: STZ-induced diabetic rats without transplantation of insulin-producing cells) Results are presented as the mean ± SD for 6 rats. ^∗^:*P* < 0.05 BM-IPCs group compared to WJ-IPCs group; ^#^: *P* < 0.05 resected pancreas-IPCs compared to WJ-IPCs group.

In our experiments, the fasting blood glucose levels in STZ-induced diabetic rats were >350 mg/dL for one week and then increased to >400 mg/dL. The fasting blood glucose levels after STZ induction in the 4 groups were not the same, though they were not significantly different. In order to further normalize the post-STZ blood glucose level, we reported the change of fasting blood glucose levels between the specific time point and the day after STZ induction instead of the exact fasting blood glucose levels at the specific time point. The trends of blood glucose level of the three study groups after IPCs transplantation were all significantly different from the control group which was consistent with our previous study results. In the WJ-IPCs group, blood glucose levels were significantly reduced since the first week after transplantation, reached the maximal decrease at week 2, and maintained significant decrease till the end point of this study, 8 weeks after transplantation. In the BM-IPCs group, blood glucose levels were also significantly reduced since the first week after transplantation, reached the maximal decrease at week 2, but gradually increased since week 3. In the resected pancreas-IPCs group, blood glucose levels were significantly reduced till two weeks after transplantation, reached the maximal decrease at week 3, and gradually increased since week 4 (Figure 4).

### 3.5. Immunofluorescence Analysis of Pancreas in Rats after Transplantation of IPCs Derived from Resected Human Pancreatic Tissue, BM-MSCs, and WJ-MSCs

Groups of cells expressing green fluorescent protein (GFP) and human C-peptide were detected in the pancreas of STZ rats 8 weeks after transplantation. We successfully found that the WJ-IPCs had homing capacity to the pancreas. However, we could not find any IPCs in the pancreas of STZ rats which received transplantation with BM-IPCs and resected pancreas-IPCs.

## 4. Discussion

Since the shortage of organ donors has hampered the progress of pancreas transplantation as well as islet transplantation, alternative sources of insulin-producing cells are mandatory to overcome this hurdle. Stem cell regeneration has become a potential insulin replacement therapy. Stem cells from the pancreas [13–16], bone marrow [12, 17], umbilical cord blood [18], and embryo [19] have been used in research on regeneration therapies for DM.

Islet-like endocrine precursor cells (EPCs), one of the cell sources for regeneration therapy, are believed to exist either in pancreatic duct cells or in the islets themselves [20, 21]. The pancreas is composed of endocrine and exocrine compartments [22]. The endocrine compartment consists of *α*, *β*, *δ*, and pancreatic polypeptide cells, which are made up of islets, whereas the exocrine compartment contains acinar and ductal cells. Several studies have disclosed an interesting insight into the potential capability of the ductal cells to generate islet-like endocrine precursor cells, which develop into islet cells [23–28]. The islet-like endocrine precursor cell is thought to be one of the sources of new islet cells in adulthood [29].

MSCs, another type of promising stem cells, were first isolated from BM [30] and were found to have the potential to differentiate into different cell lineages, not only pancreatic *β* cells [10, 31], but also muscle cells, adipocytes, osteocytes, chondrocytes [32, 33], and cardiomyocytes [23–26]. They can be incorporated into a variety of tissues, including bone [27, 28], muscle [34], lung [34], and epithelium [35] following systemic injection. Studies have shown that IPCs can be developed from BM-MSCs [36], adipose tissue-derived MSCs [37], and human umbilical cord blood-derived mononuclear cells [18], which suggest their potential for use in autotransplantation. Recently, researchers have focused on isolating and cultivating the islet-like endocrine precursor cells in both fetal and adult pancreatic tissues [38, 39].

McElreavey et al. [40] reported the isolation of fibroblast-like cells from Wharton's jelly of the human umbilical cord, which are similar to BM-MSCs [41]. Researchers endeavored to obtain, characterize, and evaluate WJ-MSCs both* in vitro* and* in vivo*. The WJ-MSCs held many unique features of mesenchymal stromal cells such as the well-known surface phenotype, the plastic adherence, and the multipotency [42–45]. In fact, in appropriate* in vitro* stimulation, they could differentiate into adipocytes, osteoblasts, chondrocytes, hepatocytes, and cardiac and neural cells. They were considered to be ready for a new source for therapeutic materials [10].

In previous studies of our research team, insulin-producing cells were generated from Wharton's jelly, bone marrow, and human pancreatic tissues. Our cultivation and differentiation protocols referred to both predecessors in the literature and editors of the journals to which we submitted our reports. Owing to the distinct cell sources, different cultivation and differentiation protocols were applied. Nonetheless, we believed that the insulin-producing cells we generated from the three different sources were in their optimal condition after our specific differentiation methods. Next, we tried to investigate the cells'* in vitro* ability to secrete insulin and the* in vivo* curative effects of the transplanted cells in diabetic rats.

Human pancreatic stem cells* in vitro* seem to have the highest insulin/DNA ratio and are most affected by high glucose stimulation. However, these cells seemed to have a low success rate in the culture medium and difficulty in growing. In the present study, the successful culture rate of surgically resected pancreatic tissue was 35% and the differentiation potential was only one passage. Unsurprisingly, similar reports are found in the literature [46–48]. In embryonic development, insulin-producing beta cells were suspected to be engineered from the endocrine precursor cells existing in the pancreatic tissue, including the exocrine part, the ductal cells, and the endocrine part, the islet cells [49, 50]. The current consensus of scholars is that there are still certain obstacles to determine whether the potential plasticity of pancreatic tissue can serve as a source of new *β* cells [51, 52]. Our data also support this point of view.

Regarding the MSCs from BM and Wharton's jelly, the former expressed positive C-peptide staining on day 14 and the latter had greater staining on day 10. In addition, the BM-MSCs had differentiation potential of 4–6 passages whereas the WJ-MSCs had differentiation potential of 8–10 passages. Moreover, when at low glucose concentration, the human C-peptide levels in WJ-MSCs were higher than those of both BM-MSCs and pancreatic MCSs. Our data showed evidence similar to the literature [53, 54] that the WJ-MSCs have a certain degree of superiority than BM-MSCs.

In the* in vivo* exam, the three kinds of insulin-producing cells were transplanted into the livers of STZ-induced diabetic Sprague Dawley rats and the therapeutic effects of transplantation were evaluated. The STZ-induced diabetic rats were hyperglycemic with blood glucose results over 350 mg/dL for one week, which increased up to more than 400 mg/dL. In the Wharton's jelly MSCs treatment group, blood glucose levels returned to nearly normal levels one week after transplantation (<200 mg/dL for 6 weeks). In the BM- MSCs group, blood glucose levels also showed a significant decrease one week after transplantation (<250 mg/dL for 5 weeks). In the pancreatic IPCs group, blood glucose levels started to decrease three weeks after transplantation and were only maintained for 4 weeks at about 300 mg/dL. Based on these results, WJ-MSCs seemed to possess greater therapeutic potential than the other two cell sources.

In conjunction with the blood glucose level and the potential role of the transplanted IPCs, histological analysis after transplantation should be performed. In our previous research, we had used immunohistochemical analysis to detect the IPCs we transplanted into the STZ rat via portal vein. We had found the appearance of both human C-peptide and human nuclei labeled BM-MSCs in the liver of STZ rat 8 weeks after transplantation therapy [12]. Human C-peptide-positive cells had been detected within the liver tissue of the STZ rat 6 weeks following WJ-MSCs transplantation [11]. C-peptide expressing resected pancreas EPCs grafts were located in the STZ rat liver 9 weeks after transplantation [13]. All of the above findings suggested that the three kinds of IPCs could be functional on ameliorating hyperglycemia and capable of survival at liver after transplantation via portal vein. In this study we make efforts trying to look for whether the IPCs would engraft other places. Since groups of cells expressing both GFP and human C-peptide were detected only in the pancreas of STZ rats who received WJ-IPCs transplantation, we successfully manifested that the WJ-IPCs had homing capacity to the pancreas. Accordingly, we recommended that WJ-IPCs were better than the other two cell sources because of their superior potential for homing to the impaired pancreas tissue and providing regenerative effects in the surrounding niche.

Wharton's jelly was used by investigators in a previous study as opposed to pancreatic stem cells because it contains many times more stem cells than the pancreatic duct [8]. Additionally, WJ-MSCs can be easily isolated and expanded in culture with a higher frequency of colony-forming fibroblasts and shorter population doubling time [40]. The generation of large amounts of functional islets is an important step for the success of regeneration therapy for diabetic patients. To summarize the results of our* in vitro* experiments, it is reasonable to conclude that the WJ-MSCs were superior proliferation and differentiation potential to both the BM-MSCs and the pancreatic MSCs. In addition, Wharton's jelly stem cells are preferable to embryonic stem cells, which were thought to have excellent differentiation potential into insulin secreting cells since using them avoids the risk of forming teratomas as well as the ethical issues inherent in using embryonic stem cells.

In conclusion, our results show that WJ-MSCs can differentiate into pancreatic lineage cells* in vitro* and function as insulin-producing cells both* in vitro* and* in vivo*. These results suggest that WJ-MSCs are a promising cell source for regeneration therapy in diabetes. Further study is required to examine the curative effects of WJ-MSCs in larger diabetic animal models and to apply clinically in human beings.

## Figures and Tables

**Figure 5:**
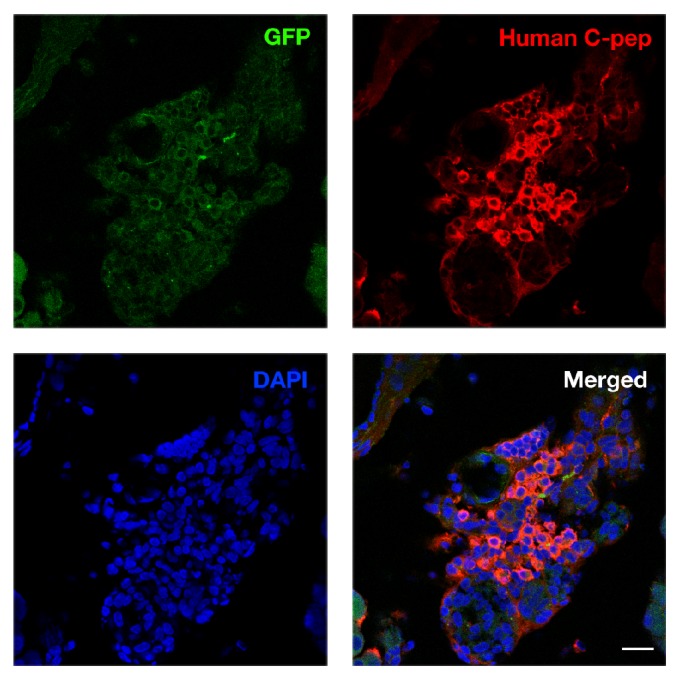
WJ-MSCs differentiated into IPCs and resided in the pancreas of the STZ rat. Confocal analysis of the pancreas tissues 23 days after WJ-IPCs transplantation showed clusters of cells with GFP expression and human C-peptide expression (red) as well as the nuclear stain, DAPI (blue). Scale bar = 50 *μ*m.
